# Parental Encouragement of Healthy Lifestyles for Their Children and Personally Caring about Healthy Lifestyles Is Positively Associated with Children Using Vitamin D Supplements

**DOI:** 10.3390/nu8100596

**Published:** 2016-09-24

**Authors:** Lalani L. Munasinghe, Yan Yuan, Erin L. Faught, Noreen D. Willows, Paul J. Veugelers

**Affiliations:** 1 School of Public Health, University of Alberta, Population Health Intervention Research Unit, 3-50 University Terrace, 8303 112 Street, Edmonton, AB T6G2T4, Canada; lalani.m@ualberta.ca (L.L.M.); efaught@ualberta.ca (E.L.F.); 2 School of Public Health, University of Alberta, 3-299 Edmonton Clinic Health Academy, 11405 87 Avenue, Edmonton, AB T6G1C9, Canada; yan.yuan@ualberta.ca; 3 Agricultural, Food & Nutritional Science, University of Alberta, 4-378 Edmonton Clinic Health Academy, 11405 87 Avenue, Edmonton, AB T6G1C9, Canada; nwillows@ualberta.ca

**Keywords:** vitamin D, child, dietary supplements, parenting, behavior

## Abstract

Supplement users have better vitamin D status, and parenting is key to promoting a child’s healthy behaviours. We examined the association of parental encouragement of and caring about healthy lifestyles with children’s use of vitamin D supplements and multivitamins. A provincially representative sample of grade 5 students (*n* = 2686; 10–11 years) and their parents across the province of Alberta, Canada, was surveyed in 2014. Students were asked about use of multivitamins and/or vitamin D supplements. Parents were asked whether they cared about and encouraged healthy lifestyles. Mixed effect multiple logistic regression identified the association of parental responses with children’s use of supplements; 29% and 54% of children took vitamin D supplements and multivitamins, respectively. They were more likely to take vitamin D supplements if their parents cared ‘very much’ vs. ‘not at all/a little bit’ about eating healthy foods (OR = 1.43; 95% CI = 1.08, 1.89), cared ‘quite a lot’ (OR = 1.55; 95% CI = 1.17, 2.04) and ‘very much’ (OR = 1.67; 95% CI = 1.26, 2.21) vs. ‘not at all/a little bit’ about physical activity, and encouraged ‘very much’ vs. ‘not at all/a little bit’ their children to eat healthy foods (OR = 1.51; 95% CI = 1.05, 2.17). Children whose parents personally cared for eating healthy foods were more likely to take multivitamins (‘quite a lot’ and ‘very much’ compared to ‘not at all/a little bit’ (OR = 1.60; 95% CI = 1.13, 2.28 and OR = 1.46; 95% CI = 1.04, 2.06, respectively). Education and parental encouragement of healthy lifestyles should be part of the public health initiatives to promote supplementation of vitamin D among children.

## 1. Introduction

The 2012/2013 Canadian Health Measures Survey [1] revealed that nearly 22% of Canadian children aged three to 11 years old have serum vitamin D concentrations that are potentially at risk of “inadequacy” or “deficient” relative to bone health. Vitamin D sufficiency is associated with supplement use among Canadians [2,3,4,5,6] and studies worldwide [7,8] have suggested supplementation with vitamin D as a strategy to reduce poor vitamin D status. Despite the evidence that Canadians who use supplements are more likely to maintain adequate vitamin D levels [3,4], it has been estimated that 71% of grade 5 children in the province of Alberta do not take vitamin D supplements [9]. Health Canada, which is the department of the federal government responsible for helping Canadians maintain and improve their health, only recommends daily vitamin D supplements for breastfed, healthy term infants and those over 50 years of age [10] despite the evidence that vitamin D supplementation seems to play an important role in achieving vitamin D adequacy in other age groups as well.

Parents are in a key position to encourage healthy behaviors among their children [11,12,13,14,15,16]. Studies have identified positive associations of parental influence on their young children’s physical activity [12,13,14,15,16], healthy eating [11,15,16] and vegetable and fruit consumption [17], such that parental perceptions regarding healthy behaviours for children were also found to be associated with children’s healthy body weight [11,14,15]. Although the effectiveness of the influence of parenting on supporting their children’s lifelong healthy behaviours wanes as the child matures [12,16], both physical activity and healthy eating of toddlers, pre-schoolers, young children and adolescents are influenced by parental encouragement or behavior [16]. The inconsistent results showed the parental influence on children’s physical activity would vary with the gender of the child and the parent [12,13]. To our knowledge, no study has been conducted addressing the association between supplement use by children and parental support of healthy lifestyle behaviours for their children or parental health practices.

The purpose of this study was to examine the influence of parental encouragement of and caring about healthy lifestyles on children’s use of vitamin D supplements and multivitamins. This knowledge can inform health promotion strategies to improve vitamin D status among Canadian children.

## 2. Methods

### 2.1. Study Design and Subjects

We analyzed demographic, socio-economic, dietary, supplement use, and physical activity data that were collected in the spring of 2014 as part of the “Raising healthy Eating and Active Living Kids in Alberta” (REAL Kids Alberta) survey. The REAL Kids Alberta survey is a population-based study of grade five students (age 10–11 years) and their parents or guardians throughout the province of Alberta, Canada. The sampling frame included 90.2% of all elementary schools in Alberta with grade 5 students. Excluded were the 9.8% of students attending francophone schools, on-reserve federal schools, and private, charter and colony schools [18]. A total of 140 elementary schools were randomly selected from three geographical strata (metropolitan, urban, and rural) to achieve proportional representation [18]. More details on the project’s aim and the measures used are available on the project’s website: http://www.realkidsalberta.ca. A total of 4993 parent surveys and parental consent forms were handed out to students to be completed by parent(s) or guardian(s) at home. Of the 3284 home consent forms returned to school (66%), 2958 students (90%) were granted parental consent and participated in the study resulting in an overall participation rate of 59%. Observations of students who had not completed the food frequency questionnaires (*n* = 107) or who had missing data on use of multivitamins and/or vitamin D supplements use (*n* = 46) were excluded from the analysis. A total of 119 students who had reported energy intakes of <500 kcal or >5000 kcal were also excluded as per established criteria when food frequency questionnaire data are involved [19]. Final analysis of the present study was restricted to a total of 2686 students (53.8%).

### 2.2. Assessment of Outcome Measures: Vitamin D-Containing Supplement Use

Each student completed a modified version of the Harvard Youth/Adolescent Food Frequency Questionnaire (FFQ) on a school day with the guidance of a trained evaluation assistant. The FFQ has been validated for use in children and adolescents aged 9 to 18 years [20]. The FFQ was modified to collect information on the use of vitamin D supplements and multivitamins by including the questions “Do you take vitamin D supplements (pills/drops)?” and “Do you take multivitamins?” These questions were reviewed by a panel with expertise in nutrition and piloted in the target population to ensure their comprehension of the questions. The commonly available multivitamin supplements for school children in Alberta contain vitamin D (informal survey of over-the-counter supplements). In this study we define “Vitamin D supplement users” as children who reported ever taking a vitamin D supplement in the past year and “multivitamin supplement users” as children who reported ever taking a multivitamin in the past year.

### 2.3. Assessment of Exposures Variables

Survey questions for parents included: (1) “To what extent do you encourage your grade 5 child to eat healthy foods?”; (2) “To what extent do you encourage your grade 5 child to be physically active?”; (3) “How much do you personally care about eating healthy foods?”; and (4) “How much do you personally care about staying fit and exercising?”. The responses for each question were given as an ordinal scale from 1 to 4, where a response of 1 = “not at all”, 2 = “a little bit”, 3 = “quite a lot” and 4 = “very much”. All survey questions were reviewed by a scientific advisory committee and piloted in the target population to ensure clarity and comprehension.

### 2.4. Assessment of Potential Confounders

The potential confounders were selected based on prior studies [11,14], due to lack of data on true confounders. Parents reported both their gender and their child’s gender in the home survey. Data on parent educational attainment and household income were collected from the home survey. Evaluation assistants measured students’ weight to the nearest 0.1 kg using calibrated digital scales (Health-o-meter^®^, Alsip, IL, USA) and standing height to the nearest 0.1 cm using stadiometers (Seca-Stadiometers, Hamburg, Germany). Body Mass Index (BMI) was calculated as weight divided by height squared (kg/m^2^). Overweight and obesity were defined according to the age- and gender- specific cut-offs of the International Obesity Task Force BMI for children and youth [21]. Region of residence was defined as metropolitan (Calgary and Edmonton, cities with a population of more than one million people), urban (other municipalities with more than 40,000 residents) and rural (municipalities with less than 40,000 residents) [18]. Child physical activity level (PAL) was based on a physical activity score ranging from 0 (lowest) to 5 (highest) derived from a 29-item questionnaire adapted from the validated Physical Activity Questionnaire for Older Children [22]. Diet quality was derived using the Diet Quality Index-International based on adequacy, variety, moderation, and balance, with scores ranging from 0 representing the lowest to 100 the highest diet quality [23]. Dietary intake data were obtained based on responses to the questions in the FFQ, validated for children and adolescents [20]. Total calorie intake from food was calculated using the Canadian Nutrient File [24].

### 2.5. Statistical Analyses

The response categories of the exposure variables of interest of ‘not at all’ and ‘a little bit’ were combined into one category due to the small number of responses. Descriptive statistics were used to characterize the students and to identify the frequency of parents’ responses. Mixed effect logistic regression analysis with children nested within schools was used to examine the association of vitamin D supplement and multivitamin use among children with parental care about healthy foods and physical activity, and parental encouragement to undertake these behaviors. We first applied univariable regression analysis to quantify the unadjusted associations. Second, we applied multivariable regression analyses to adjust for the confounding effects of student gender, parental gender, parental education, household income, region of residence, body weight status, PAL and energy adjusted diet quality index. We adjusted for PAL and diet quality to quantify the association of parenting behaviors on supplement use independent of these children’s lifestyles. Third, we constructed two parsimonious regression models in which we considered both parental encouragement to eat healthy foods and to be physically active simultaneously, or in which we considered both parental personal care on those behaviors simultaneously with the potential confounders. Last, we constructed a full parsimonious regression model that considered all exposure variables simultaneously with the potential confounders to quantify their independent contribution to vitamin D—containing supplement use. Model selection was done using Akaike Information Criteria (AIC) and Bayesian Information Criteria (BIC), and the model with lowest AIC and BIC was selected. Interaction models were built to identify if parental encouragement and personal caring about healthy eating and if parental encouragement and personal caring about being physically active had any synergistic effect on the outcome of interest. As those interaction effects appeared small and not statistically significant, we do not present the results of those interaction models. Missing data for some of the confounder variables were treated as a separate category. An “energy adjusted diet quality index” was computed from the residuals of a regression model with total energy intake as the independent variable and diet quality index as the dependent variable, as per established criteria [19]. All analyses were weighted to represent unbiased provincial estimates of the grade 5 student population in Alberta. Data were analyzed using Stata version 13 (Stata Corp., College Station, TX, USA). Research Ethics Board at the University of Alberta approved all procedures involving human subjects and ethical approval was obtained for the current secondary data analysis.

## 3. Results

Characteristics of grade 5 students and their parents/guardians are presented in Table 1. The percentages were weighted to represent provincial estimates of the grade 5 student population aged 10–11 years old in Alberta. Altogether, 8.06% of the students took only a vitamin D supplement, 32.69% took only a multivitamin, and 21.39% took both a vitamin D supplement and multivitamin. This means that 29.45% took a vitamin D supplement and 54.08% took a multivitamin. The majority of the parents/guardians reported personally caring about and encouraging their children to eat healthy foods and to be physically active (Table 1). Supplementary tables (Tables S1 and S2) show the correlation between the exposures of interest, i.e., parental encouragement of and personally caring about healthy eating and being physically active.

Table 2 shows the associations between children’s use of vitamin D supplements and parental encouragement to eat healthy foods, parental encouragement to be physically active, parents personally caring about healthy eating, and parents personally caring about physical activity. Parental encouragement of and caring about eating healthy and physical activity were positively associated with vitamin D supplement use after adjusting for potential confounders. Students whose parents encouraged them ‘very much’ (OR = 1.51; 95% CI = 1.05, 2.17) and whose parents personally cared ‘very much’ (OR = 1.43; 95% CI = 1.08, 1.89) about eating healthy were more likely to use vitamin D supplements as compared to students whose parents encouraged or cared ‘not at all’ or ‘a little bit’ about healthy eating (Table 2). Students whose parents personally cared ‘quite a lot’ (OR = 1.55; 95% CI = 1.17, 2.04) and ‘very much’ (OR = 1.67; 95% CI = 1.26, 2.21) about being physically active were more likely to use vitamin D supplements as compared to students whose parents reported ‘not at all/a little bit’ (Table 2). Parental personal care about being physically active remained significant when caring about healthy eating and caring about physical activity were considered simultaneously (parsimonious model: data not shown). Likewise, parental personal care about being physically active remained significant in a parsimonious model with all four exposures of interest (parental encouragement to eat healthy and to be physically active, and parental personal care about healthy eating and being physically active). Parents personally caring ‘quite a lot’ (OR = 1.53; 95% CI = 1.14, 2.06) and ‘very much’ (OR = 1.58; 95% CI = 1.10, 2.26) vs. ‘not at all/a little bit’ about physical activity was strongly associated with vitamin D supplement use in the final parsimonious regression model adjusting for confounders and the other three exposure variables. All other associations except parental educational attainment were not statistically significant.

Table 3 depicts the association of parental encouragement of and care about healthy eating and physical activity with vitamin D–containing multivitamin supplement use. Only students whose parents personally cared ‘quite a lot’ (OR = 1.60; 95% CI = 1.13, 2.28) and ‘very much’ (OR = 1.46; 95% CI = 1.04, 2.029) about eating healthy foods were more likely to use multivitamin supplements as compared to students whose parents personally cared ‘not at all/a little bit’. In a parsimonious regression model with all four exposures of interest, only parental personal care about eating healthy foods was positively associated with vitamin D–containing multivitamin supplement use (OR = 1.55 ; 95% CI = 1.06, 2.27 for ‘quite a lot’ and OR = 1.47; 95% CI = 0.98, 2.19 for ‘very much’ relative to ‘not at all/a little bit’).

## 4. Discussion

To our knowledge, this study is the first to reveal the association of children’s use of vitamin D supplements and multivitamins with parental encouragement of healthy lifestyles and personally caring about healthy lifestyles. Specifically, in this large population-based study, we identified a positive association between parental encouragement of children’s healthy eating and personally caring about healthy eating and being physically active with child vitamin D supplement use. However, only parental caring about eating healthy foods was positively associated with children’s multivitamin use. The lack of association between parental encouragement of eating healthy foods and multivitamin use may be due to the possibility that parents who frequently encourage their children to eat healthy foods may emphasize meeting nutritional needs through healthy eating rather than through multivitamin supplements. Meanwhile, parents who encourage their children to take vitamin D supplements may understand that children cannot meet vitamin D needs through diet alone and, as such, need to take supplements. The observed increasing gradient in odds of using vitamin D supplements as parental caring and encouragement increased is indicative that even small changes in parental engagement in their children’s health behaviours can have a large impact on their likelihood of demonstrating positive healthy behaviours. Our research program had previously identified parental education as a determinant of vitamin D supplement use among children [9]. It has also revealed that both parental encouragement of children’s healthy eating and parental caring about healthy eating are independently associated with the diet quality of their children [11]. Parents who care about healthy eating may have increased availability of healthy foods in the home and model healthy behaviours, and encouragement may be important in guiding children’s choices when healthy options are available [25]. Additionally, parental caring, encouragement, and engagement in physical activity have been found to be independently and positively associated with children’s physical activity levels and negatively associated with the likelihood of the child being overweight [14]. Other studies have also revealed that positive parental influences lead to better diets and higher activity levels in children [11,12,13,14,15,16,17]. The present study demonstrates that even small increases in parental caring and encouragement of healthy behaviours can contribute substantially to the likelihood of children engaging in healthy behaviours such as the regular use of vitamin D supplements.

The diet is the best way for children to meet nutritional requirements [26,27]. However, as Canadian children have limited sun exposure and often do not consume enough vitamin D—rich foods to meet the daily requirement [28], supplementation with vitamin D will help children to achieve vitamin D adequacy and subsequent good bone health. Vitamin D supplementation, however, receives little attention as a public health priority for Canadian children and is only recommended for children under the age of one [10]. This may be why only 29% of grade 5 students in this 2014 study took vitamin D supplements. Comparatively, 29% girls and 28% boys aged six to 11 years used a vitamin D—containing supplement in a 2007/2009 Canadian national survey [29]. In the present study, we revealed that parental caring about healthy lifestyles is associated with children’s use of vitamin D supplements. Therefore, increasing parents’ awareness of the high prevalence of vitamin D insufficiency and the importance of supplementation may contribute to children’s use of supplements and, consequently, improved vitamin D status among children. We also revealed that parental encouragement of healthy lifestyles was associated with the use of supplements among children. This seems to imply that educating parents on the importance of vitamin D, in combination with promoting parental encouragement of supplement use, may increase supplement intake among children and improve their vitamin D status. However, intervention research will be needed to establish this supposition and determine the sustained effects of such an intervention on lifelong habits.

Child overweight and obesity is a serious public health challenge worldwide [30] and in our study, 29% of children were overweight/obese. Body mass index is negatively associated with vitamin D status [31,32,33,34,35] and vitamin D inadequacy is highly prevalent among children who are overweight or obese compared to non-overweight children [1]. Obese children may have elevated vitamin D requirements due to the sequestration of lipid-soluble vitamin D in a larger pool of adipose tissues [6,35,36], lower vitamin D metabolism in the liver [36], increased vitamin D catabolism in the kidneys [6,36], or limited sun exposure due to low physical activity levels [36]. The beneficial effect of healthy lifestyle practices on preventing excess body weight is widely reported [11,14,15]. Therefore, parental influence on promoting healthy lifestyle practices would indirectly improve vitamin D status among children with excess body weight, independent of the effect of parental influence on increasing supplement use among children.

Strengths of the present study include the use of a large provincially representative sample with a relatively high response rate for school-based research and the execution of multilevel regression to account for the hierarchical data structure. We accounted for the survey design effect with weighted analysis. However, non-response may cause a bias in the estimates if the missing data are not random. We considered missing data as a separate category in the regression analysis and did not find any significant association with the outcomes. This study also has several limitations. Self-report can be a cause of error, though the use of a validated FFQ and Physical Activity Questionnaire may have minimized this to some extent. In addition, non-differential misclassification of outcomes is possible if children were unable to distinguish between vitamin D supplements and multivitamins. However, evaluation assistants helped minimize this issue by providing explanations to children while they completed the questionnaires. The results may not be generalizable to all elementary school students in Alberta, Canada, due to the exclusion of 9.8% of elementary schools from the sampling frame and possible non-response from certain socioeconomic groups. However, no data is available on non-respondents to evaluate this speculation. Residual confounding is likely due to the unavailability of data on parental lifestyle factors including parental use of supplements as a potential confounder. Further, health behaviour questions are prone to social desirability bias. As both students and parents were informed about maintaining confidentiality of data before they provided consent for participation, this bias may be minimized. Lastly, caution is warranted when interpreting causality due to the cross-sectional nature of the study design.

## 5. Conclusions

Our study revealed the important role of parents for achieving adequate vitamin D intake by their children: parental encouragement of and caring about healthy lifestyles were observed to be associated with the use of vitamin D supplements and multivitamins. Less than one third of children in this study reported using vitamin D supplements despite the fact that the dietary vitamin D intake of Canadian children is often inadequate and vitamin D deficiency and insufficiency are prevalent. Public health initiatives to promote vitamin D supplement use among children should be considered. Our findings suggest that education about the need for vitamin D supplements among children and the importance of parental encouragement and caring about vitamin D as part of a healthy diet is necessary to reduce the prevalence of vitamin D insufficiency among Canadian children. Future studies investigating the long-term influence of other aspects of parenting on children’s vitamin D supplement use and the maintenance of healthy habits as children age would be beneficial to support these findings.

## Figures and Tables

**Table 1:** Characteristics of 10–11 year-old students in Alberta, Canada, and their parents’ responses *.

Characteristic	% of Students (*n* = 2686)
**Parental encouragement for children to eat healthy foods**	
Not at all/A little bit	7.12
Quite a lot	45.71
Very much	44.31
**Parental encouragement for children to be physically active**	
Not at all/A little bit	12.04
Quite a lot	42.32
Very much	42.73
**Parental personal care for eating healthy foods**	
Not at all/A little bit	13.12
Quite a lot	45.70
Very much	38.06
**Parental personal care for being physically active**	
Not at all/A little bit	21.66
Quite a lot	45.27
Very much	30.20
**Vitamin D supplements**	
Users	29.45
Non-users	70.55
**Multivitamins**	
Users	54.08
Non-users	45.92
**Child’s gender**	
Girls	53.49
Boys	46.51
**Parental gender** ^†^	
Female	80.89
Male	14.94
**Parental education** ^†^	
Secondary or less	23.19
College	33.54
University/graduate	37.89
**Household income**	
≤$50,000	13.21
$50,001–$100,000	19.03
≥$100,001	28.89
Non-disclosed/Missing ^§^	38.87
**Region of residence**	
Rural	39.55
Urban	8.28
Metropolitan	52.17
**Weight status** ^†^	
Under/normal weight	68.59
Overweight	20.84
Obese	7.86

* Results were weighted to represent provincial estimates of the grade 5 student population (age: 10–11 years old) in Alberta; ^†^ <5% of missing data for parental gender, parental education and weight status; ^§^ 26.63% non-disclosed responses (participants were provided option ‘prefer not to answer’ their household income) and 12.23% missing data.

**Table 2:** Associations of vitamin D supplement use with parental encouragement and care about healthy lifestyle practices *.

	Vitamin D Supplement Users	Univariable Model			Multivariable Model ^§^		
	% (*n* = 769)	OR	95% CI	*p* Value	OR	95% CI	*p* Value
**Parental encouragement for children to eat healthy foods** ^†^							
Not at all/A little bit	5.34	1.00			1.00		
Quite a lot	43.53	1.37	0.97, 1.94	0.076	1.31	0.91, 1.90	0.149
Very much	48.17	1.65	1.17, 2.33	**0.004**	1.51	1.05, 2.17	**0.025**
**Parental encouragement for children to be physically active** ^†^							
Not at all/A little bit	10.06	1.00			1.00		
Quite a lot	39.99	1.18	0.84, 1.65	0.333	1.10	0.77, 1.56	0.598
Very much	47.18	1.47	1.09, 1.98	**0.012**	1.26	0.92, 1.74	0.153
**Parental personal care for eating healthy foods** ^†^							
Not at all/A little bit	10.47	1.00			1.00		
Quite a lot	44.32	1.30	0.97, 1.73	0.080	1.25	0.93, 1.67	0.142
Very much	41.87	1.55	1.18, 2.04	**0.002**	1.43	1.08, 1.89	**0.012**
**Parental personal care for being physically active** ^†^							
Not at all/A little bit	15.85	1.00			1.00		
Quite a lot	47.09	1.60	1.22, 2.11	**0.001**	1.55	1.17, 2.04	**0.002**
Very much	34.24	1.82	1.40, 2.38	**<0.001**	1.67	1.26, 2.21	**<0.001**

* Results were weighted to represent provincial estimates of the grade 5 student population (age: 10–11 years old) in Alberta. Vitamin D supplement users were defined as those who used vitamin D supplements irrespective of use of multivitamins. ^†^ <4% of missing data; ^§^ Adjusted for student gender, parental gender, parental education, household income, region of residence, body weight status, physical activity and the energy adjusted diet quality index.

**Table 3:** Associations of vitamin D–containing multivitamin use with parental encouragement and care about healthy lifestyle practices *.

	Multivitamin Users	Univariable Model			Multivariable Model ^§^		
	% (*n* = 1468)	OR	95% CI	*p* Value	OR	95% CI	*p* Value
**Parental encouragement to eat healthy foods** ^†^							
Not at all/A little bit	5.80	1.00			1.00		
Quite a lot	45.84	1.48	1.05, 2.11	**0.026**	1.34	0.94, 1.91	0.102
Very much	46.01	1.59	1.16, 2.18	**0.004**	1.35	0.97, 1.89	0.075
**Parental encouragement to be physically active** ^†^							
Not at all/A little bit	10.73	1.00			1.00		
Quite a lot	42.06	1.26	0.91, 1.74	0.161	1.16	0.82, 1.63	0.396
Very much	44.83	1.40	1.02, 1.93	**0.039**	1.16	0.83, 1.64	0.384
**Parental personal care of eating healthy foods** ^†^							
Not at all/A little bit	10.39	1.00			1.00		
Quite a lot	47.74	1.75	1.25, 2.44	**0.001**	1.60	1.13, 2.28	**0.009**
Very much	39.22	1.68	1.23, 2.30	**0.001**	1.46	1.04, 2.06	**0.029**
**Parental personal care of being physically active** ^†^							
Not at all/A little bit	20.21	1.00			1.00		
Quite a lot	46.27	1.21	0.97, 1.51	0.094	1.15	0.92, 1.44	0.221
Very much	31.20	1.23	0.97, 1.56	0.092	1.08	0.84, 1.38	0.550

* Results were weighted to represent provincial estimates of the grade 5 student population (age: 10–11 years old) in Alberta. Multivitamin users were defined as those who used multivitamins irrespective of use of vitamin D supplements. ^†^ <3% of missing data. ^§^ Adjusted for student gender, parental gender, parental education, household income, region of residence, body weight status, physical activity and the energy adjusted diet quality index.

## Supplementary Materials

The following are available online at http://www.mdpi.com/2072-6643/8/10/596/s1, Table S1: Prevalence between parents’ responses to their encouragement for and care about eating healthy foods, Table S2: Prevalence between parents’ responses to their encouragement for and care about being physically active.
