# Supplementary material for: Parental Encouragement of Healthy Lifestyles for Their Children and Personally Caring about Healthy Lifestyles Is Positively Associated with Children Using Vitamin D Supplements
Source: Nutrients. 2016 Sep 24;8(10):596. doi: 10.3390/nu8100596 (PMC5083984; doi:10.3390/nu8100596)
Supplement: Supplementary file 1 [file nutrients-08-00596-s001.pdf]

Supplementary Materials: Parental Encouragement of Healthy Lifestyles for Their Children and Personally Caring about Healthy Lifestyles Is Positively Associated with Children Using Vitamin D Supplements

Lalani L. Munasinghe, Yan Yuan, Erin L. Faught, Noreen D. Willows and Paul J. Veugelers

Table S1. Prevalence between parents' responses to their encouragement for and care about eating healthy foods *.

	Parental Personal Care for Eating Healthy Foods		
	Not at all/A little bit (%)	Quite a lot (%)	Very much (%)
**Parental encouragement for children to eat healthy foods**			
Not at all/A little bit	28.66	4.32	3.46
Quite a lot	57.65	63.60	22.97
Very much	12.23	31.91	73.24

* Results were weighted to represent provincial estimates of the grade 5 students population (age: 10–11 years old) in Alberta. Missing data is <2%. p-value for Pearson Chi-sq test < 0.001.

Table S2. Prevalence between parents' responses to their encouragement for and care about being physically active *.

	Parental Personal Care about Being Physically Active		
	Not at all/A little bit (%)	Quite a lot (%)	Very much (%)
**Parental encouragement for being physically active**			
Not at all/A little bit	27.67	10.22	4.55
Quite a lot	48.89	50.12	29.17
Very much	22.56	39.11	66.01

* Results were weighted to represent provincial estimates of the grade 5 students population (age: 10–11 years old) in Alberta. Missing data is <1%. p-value for Pearson Chi-sq test < 0.001.
